# Alveolar Abscess,—Treatment and Cure

**Published:** 1861-04

**Authors:** W. H. Shadoan


### ALVEOLAR ABSCESS—TREATMENT AND CURE.

BY W. H. SHADOAN.

The successful treatment of an alveolar abscess has, by many, been considered one of the impossible things; but the many experiments by scientific Dentists show that to be erroneous. I do not say that all abscesses can be cured, but I do say that many, if properly treated, can; and when I make this assertion, I do so from personal experience in a practice of about five years, which gives me the satisfaction of knowing to a certainty that alveolar abscess may be cured. How was it a few years ago with the teeth when a gum boil was occasioned by a tooth? The first thing was to say, "Oh, that tooth can't be cured, it's gone too far;" if you would have saved the tooth you should have come before the "gum [illegible] made its appearance, and had the tooth plugged.

Well, now, that all of our patients do not know what to do, or what they can do; we must take them as we find them, and if possible, save them. I remember that in a conversation with a Dr. D., of Kentucky, a few months ago, the subject of diseased nerves of the teeth and alveolar abscesses came up, and he emphatically said, that the successful treatment of either was a humbug, and that he did not believe it could be done. He said that as a proof of the assertion, that one of Dr. Watt's brag jobs of "fang filling" came to him a short time before, and that he undertook to treat it and had failed, and that he never did have success in any case, however simple. Now in fang filling, when there is alveolar abscess—we don't pretend to say we can save all cases, but we do most positively say that we can cure most cases if they are presented before the disease runs too long. For a physician to say that he can cure all his patients, under any and all circumstances, is the best proof that he is a quack and wishes to deceive.

The subject of treating alveolar abscess is not the subject of so much thought and consideration in our Dental Associations as it should be, nor is it written on so much as other subjects, perhaps of less interest than it: therefore I would suggest that some of our professional brethren should devote a portion of their time to that of the different diseases of the processes, and say less about Dental Ethics, etc.; I have no fault to find with their writings, but would like to see other subjects properly attended to.

As to the causes of alveolar abscess, I presume the profession is familiar, it being produced in most cases by acute inflammation of the Dental periosteum; sometimes caused by dead teeth, or the suppuration of the lining membrane of the pulp, or from an accumulation of purulent matter at the extremity of the tooth, the egress of which has been prevented by the natural opening being closed up. Or it may be caused by mechanical violence or some other irritating cause.

We have in a very brief manner glanced at the causes, etc., of the disease. I will now notice some of the results, with the most popular treatment, and more especially that which I adopt in my own practice, giving at the same time a few cases, showing the result. I shall give the most aggravated cases in my practice, so that you may see the success.

The final result of alveolar abscess is so well known at this day, that the time will be best spent by passing to the treatment at once. My practice was, when I first began the treatment of such cases, to use nitrate silver and creosote principally, sometimes adding a little tannin and sulphate copper. The treatment which I now adopt is chloride zinc, as a disinfectant, after which I use creosote, sometimes tinct. myrrh, sulphate copper, sulphate zinc, nitrate silver, and sugar of lead, &c., &c. My principal remedy is the first two.

I will now give a few cases to show the success I met with.

Case 1. J. H. D. B., æt. 36.—Sanguine bilious, of sedentary habit, called to have some teeth filled, one of which had as he said, a "gum boil on it." After filling all but the one to be treated, I examined it and found that the cavity of decay did not extend to the pulp cavity; I filled the cavity and treated the tooth externally—the tooth in question was the right superior first molar. Treatment: drilled a hole into the apex of the labial anterior fang from which the pus proceeded. Treated with nitrate silver and creosote, May 17, 1858.

May 19. Treated as before, patient doing well.

May 21. Patient doing very well—abscess nearly well. Directed him to use some slight purgative. In a few days he called again, perfectly cured.

Case 2. April 8, 1859. S. V. C., æt. 18.—Case of disease of the fang of the left lateral incisor, which had first the appearance of periostisis, which resulted in an abscess; formed an opening through the canal and treated as in Case 1, adding Tefft's Anæsthetic. Cured in six days.

Case 3. June 18, 1860. C. C. R.—Abscess of the anterior fang of the first right inferior molar. The pus escaped at the margin of the gum about the center of the tooth. Dr. —, of Bloomington, Indiana, had filled the tooth with amalgam four years ago, when it caused so much trouble that the filling was taken out. After that another celebrated D. D. S. of the said B., tried his hand on the tooth, and to better the case he filled with tin. The tooth came to me on June 18th, I drilled down into the cavity as far as I could, (the cavity taking a turn which prevented the successful drilling of it). I treated for several days with chloride of zinc and creosote alternately. I could cure the tooth and abscess as far as I could reach, but could not reach the seat of the disease. If the patient had not left, I should have continued the treatment, endeavoring to reach the seat, if possible. This I consider a failure for want of time.

Case 4. Mrs. R. G. called January 16, 1861.—Abscess formed at apex of fangs of first superior molar, right side—tooth filled with tin ten years ago. Removed filling, and drilled through into pulp cavity, some pus discharged, then drilled through the fangs into process, found the pus in quite a quantity. Syringed the cavity with warm water, then with a solution of chloride of zinc, and saturated small pledget of cotton with creosote, stopped up the pulp cavity and ordered a cathartic.

January 17. Patient called, doing reasonably well, soreness partially relieved, bowels in a good condition.

January 18, 9 A. M. Patient doing well, rested easy since 3 A. M., soreness all out of the tooth, the discharge ceased.

January 19, 9 A. M. Syringed tooth with warm water, treated with zinc and creosote as before. From all indications the cure is effected, there being no soreness or discharge.

January 21, 9 A. M. Removed the cotton and found the tooth well; there is no soreness or any sign of disease.

January 23, 9 A. M. Cleaned tooth as well as I could, then saturated a small pledget of cotton for each fang, and placed them in the bottom of cavity, and filled fangs with gold.

January 31, 9 A. M. Patient called that I might know how case is doing; all well, no trouble whatever.

February 6. Tooth still doing well, and since then has been no trouble; it is presumable there will be no return. Filled crown cavity with J. B. Dunlevy's gold foil.

February 13. Patient called that I might know how case is doing. A little soreness once for an hour or so, on the 10th. No indications of a recurrence since.

March 7. Case 4, doing well, patient well pleased with the success. Thinks the price of such a job rather high for one tooth, but don't complain.

